# The Role of Hydration in Children and Adolescents—A Theoretical Framework for Reviewing Recommendations, Models, and Empirical Studies

**DOI:** 10.3390/nu17172841

**Published:** 2025-08-31

**Authors:** Marek Zborowski, Magdalena Skotnicka

**Affiliations:** 1 The Faculty of Medicine and Health Sciences, University of Applied Sciences in Nowy Sącz, Kościuszki 2G, 33-300 Nowy Sącz, Poland; mzborowski@ans-ns.edu.pl; 2 Department of Commodity Science, Faculty of Health Sciences, Medical University of Gdansk, 7 Debinki Street, 80-210 Gdansk, Poland

**Keywords:** hydration status, adolescents, fluid intake, health education, dehydration

## Abstract

Proper hydration is essential for maintaining homeostasis and the effective functioning of physiological systems, including the nervous and circulatory systems. During adolescence, a period characterized by rapid somatic growth, hormonal maturation, and increased physical and mental activity, the demand for water increases significantly. Hydration affects not only the health of young people, but also their cognitive abilities, concentration, mood, and general well-being. Despite clear recommendations from institutions such as EFSA and IOM regarding daily fluid intake, numerous studies indicate that a significant proportion of young people do not achieve the recommended level of hydration. The school environment is particularly worrying, as young people spend a significant part of their day there, and the availability of water, health knowledge, and social conditions may contribute to dehydration or promote unhealthy choices (e.g., sweetened drinks). The aim of this article is to review the current state of knowledge on the importance of hydration in school-age adolescents. The physiological basis of hydration, the impact of insufficient fluid intake on the functioning of the young body, current guidelines, as well as the results of selected epidemiological studies and obstacles to ensuring optimal hydration in the school environment are discussed.

## 1. Introduction

Proper hydration is essential for maintaining health, as well as physical and mental performance. Water plays a critical role in metabolic processes by supporting biochemical reactions, facilitating nutrient transport, regulating body temperature, and aiding in the elimination of harmful metabolites [1,2,3,4]. Although water is recognized as an essential nutrient, its role in public health strategies targeting adolescents is often underestimated or overlooked [5,6,7,8,9]. Therefore, there is a clear need to raise public awareness of the widespread challenges related to hydration in this population group [10].

Adolescence is a period marked by significant physical, social, and emotional changes, all of which may pose challenges and obstacles in the life of a young individual. It is widely recognized as one of the most profound biological transformations in the human life course, during which the developing central nervous system becomes particularly sensitive to environmental factors, including water deficiency [11,12]. Adolescence is marked by increased vulnerability to dehydration due to biological, behavioral, and environmental factors, such as hormonal changes, high physical and mental demands, and insufficient awareness of fluid needs [11,12,13,14]. Even mild dehydration (1–2% loss of body mass) may negatively affect cognitive function and emotional regulation [15,16,17]. Despite well-established recommendations from institutions such as EFSA (European Food Safety Authority), IOM (Institute of Medicine), and AAP (American Academy of Pediatrics) [18,19,20,21], many adolescents do not meet daily fluid intake requirements [22,23,24]. Contributing factors include limited access to water at school, peer influence, advertising, and the widespread availability of sugar-sweetened and energy drinks [25,26,27,28,29,30,31,32,33]. These behaviors not only reduce hydration but increase the intake of caffeine and added sugars, raising the risk of obesity, hypertension, and sleep problems. In this context, a comprehensive integrative review is warranted to organize current knowledge and identify key obstacles to adequate hydration among school-age youth, particularly within the school environment. In light of these findings, a comprehensive literature review aimed at systematically organizing and interpreting current knowledge on adolescent hydration is warranted. This integrative review aims to synthesize interdisciplinary knowledge on adolescent hydration by summarizing the physiological basis of fluid needs during puberty, epidemiological evidence of insufficient fluid intake and its health consequences, and strategies for improving hydration through educational interventions and school infrastructure. The scope of the review includes literature from Europe, North America, and Asia, providing a comprehensive perspective. Importantly, recent findings in adult populations suggest that chronic low-grade dehydration may contribute to kidney dysfunction, cardiometabolic disease, and accelerated biological aging [25]. These long-term risks reinforce the importance of prevention and support the need for focused research on adolescents.

## 2. Physiological Foundations of Hydration Status and Its Regulation

### 2.1. Water Content and Roles in the Human Body

**Figure 1:**
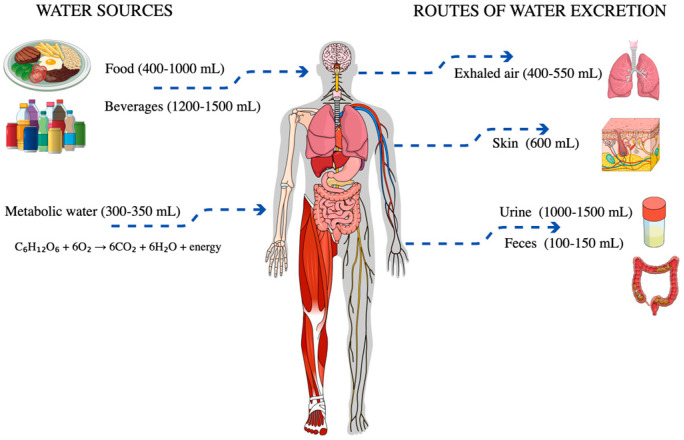
Daily loss and gain of fluids in the human body.

Water accounts for 60% to 75% of children’s and adolescents’ body mass, with its proportion decreasing with age and increasing body fat content [24,26]. During adolescence, water requirements rise due to accelerated growth, metabolic and hormonal changes, and increased physical activity [27,28]. Water plays a crucial role in thermoregulation, nutrient transport, waste elimination, plasma volume maintenance, and blood pressure regulation [3,14,29,30,31]. Water is excreted primarily through urine, but also via the skin (perspiration), lungs (respiration), and the gastrointestinal tract (Figure 1).

### 2.2. Definitions and Assessment of Hydration Status

Maintaining water balance is a complex physiological process involving both behavioral and neurohormonal mechanisms. Hydration status refers to the balance between water intake and loss. Under normal physiological conditions, euhydration indicates adequate total body water. Hypohydration describes a gradual or chronic reduction in total body water, typically due to insufficient fluid intake or increased fluid loss, which is accompanied by chronic activation of water conservation mechanisms. In contrast, dehydration refers to an acute and dynamic process in which fluid loss exceeds intake over a short period of time [26,34,35]. Hydration status can be assessed using biomarkers derived from urine and blood samples. Common urinary markers include osmolality, specific gravity, color, and volume. Urine osmolality values above 800 mOsm/kg or dark urine color often suggests inadequate hydration. Among blood markers, plasma osmolality is considered the gold standard, indicating dehydration. Additional markers such as serum sodium and hematocrit may also provide indirect evidence of hydration status. While no single biomarker is entirely sufficient, using a combination of indicators enhances assessment accuracy, particularly in research and clinical settings [18,19,34].

### 2.3. Neurohormonal Regulation of Water Balance

The antidiuretic hormone AVP (arginine vasopressin) is secreted by the posterior pituitary in response to increased plasma osmolality and promotes water reabsorption in the kidneys by acting on V2 receptors, which leads to urine concentration and reduced water loss [3,33]. The renin–angiotensin–aldosterone system (RAAS), triggered by reduced blood volume or blood pressure, contributes to vasoconstriction and sodium retention, indirectly supporting water conservation [36]. In contrast, atrial natriuretic peptide (ANP), secreted in response to atrial stretch, promotes sodium and water excretion and lowers blood pressure, counterbalancing the effects of RAAS [37]. Osmoreceptors and baroreceptors detect changes in plasma osmolality and blood pressure and initiate appropriate hormonal responses, while also stimulating thirst [3,14]. Additionally, sex hormones such as estrogen and progesterone may modulate AVP sensitivity, leading to cyclic fluctuations in water retention among adolescent females [36,38,39]. In this age group, compensatory mechanisms such as thirst perception and renal water conservation may be less efficient than in adults, resulting in more pronounced fluctuations in hydration status and increased vulnerability to dehydration, particularly during physical exertion or heat exposure [14,28,40].

## 3. Hydration in Adolescents: Physiology, Guidelines, and Health Effects

### 3.1. Physiology and Health Effects of Hydration in Adolescents

In moderate climatic conditions, the average daily water loss in adults is approximately 2 to 2.5 L. However, among children and adolescents, especially those who are physically active, water loss can be significantly higher [26]. It is important to note that children and adolescents are more susceptible to water imbalance than adults. They have a relatively higher body surface area in proportion to body mass, overheat more rapidly, and lose water more quickly during physical activity or in hot environments [14,28,40,41]. Notably, the thirst mechanism in youth is not fully efficient. They often do not feel the need to drink, even when their bodies require fluids [42]. As a result, adolescents rarely consume water preventively; instead, they tend to drink only after early symptoms of dehydration appear. These symptoms are often nonspecific and may include headache, drowsiness, irritability, and decreased concentration [15,16,43]. An increasing number of studies confirm that even mild dehydration may impair cognitive performance, particularly, working memory, reaction time, and attention span, and may cause both mental and physical fatigue [16,44,45]. For example, a study conducted by Edmonds [46] among school-aged children demonstrated a dose–response relationship between water intake and visual attention in both children and adults. Visual attention was enhanced even with small amounts of fluid, and this effect appeared to be independent of thirst reduction. The consumption of 300 mL of water improved cognitive performance within just 20 min. Similar observations were reported by Drozdowska [14], whose study, designed as a cluster-randomized controlled trial, showed that consuming up to 1000 mL of water (approximately 50% of total daily fluid intake) brought measurable benefits during memory tasks in the studied group of children. Her findings suggest that water-friendly environments support adequate hydration among school-aged children, which in turn translates into improved cognitive function, particularly short-term memory. Dehydration also affects physiological thermoregulation mechanisms, increasing the risk of overheating and thereby impairing physical performance, especially during school physical education classes or recreational activities [3,34]. Students who are inadequately hydrated more frequently report symptoms such as dizziness, fatigue, low energy levels, and difficulties with concentration, all of which directly affect their academic performance and social functioning [3,24,35]. In the school environment, inadequate hydration has multidimensional consequences, ranging from short-term decline in attention to long-term health risks. Unfortunately, despite a growing body of scientific evidence, this issue is still insufficiently addressed in school practice. Schools often lack regulations regarding access to drinking water, students are not in the habit of drinking water regularly during lessons, and sugar-sweetened beverages (SSBs) continue to dominate as the primary source of fluids. Proper hydration is a key factor in the somatic, mental, and social development of young people. Integrating this aspect into school-based prevention efforts, nutrition programs, and educational interventions appears to be a fundamental measure from a public health perspective [14,47,48].

### 3.2. Description of Adolescence and Vulnerability to Dehydration

Adolescence, defined as the age range between 10 and 19 years, represents a unique developmental stage characterized by intense somatic, neurohormonal, and psychosocial transformations [11,49]. These changes are associated with increased physiological demands, including heightened water requirements. During this period, proper hydration becomes critical not only for maintaining fluid–electrolyte homeostasis but also for supporting rapid developmental processes and cognitive function. At the same time, adolescents exhibit a number of physiological and behavioral characteristics that make them particularly vulnerable to dehydration [22,50]. A study by Aphamis [13], conducted among 102 adolescent boys aged 15 to 17 years, found that nearly 90% of participants arrived at school in a dehydrated state in the morning. Students who were significantly dehydrated (n = 50) reported feeling less alert in the early hours (*p* < 0.035), while perceived thirst levels were similar across all study groups. The frequency of dehydration during school hours appeared to be remarkably high, and thirst alone did not motivate students to consume an adequate amount of fluids.

### 3.3. Review of Hydration Guidelines and Recommendations for Adolescents

Water is the only nutrient for which daily requirements are measured based on volume rather than per unit of body mass. In the context of adolescents who are undergoing intense physiological, neurohormonal, and emotional development, determining optimal reference values for fluid intake must take into account dynamic changes in lean body mass, physical activity levels, ambient temperature, and overall dietary structure. EFSA [18] and IOM [19] provide the following recommended daily water intake levels for adolescents (Table 1).

The World Health Organization (WHO) does not publish specific reference values for daily water intake but refers to the recommendations of EFSA and IOM, emphasizing that adolescents’ hydration needs should be assessed individually- taking into account chronic illnesses, physical activity levels, and climatic conditions. The American Academy of Pediatrics [20] highlights the importance of “functional hydration,” meaning fluid intake tailored to the time of day and physiological context (e.g., exercise, illness, fever). In Poland, the National Institute of Public Health—NIH (formerly the Institute of Food and Nutrition) outlines the following water intake recommendations in the 2024 Nutritional Standards (Table 2) [21].

## 4. Social and Environmental Determinants of Beverage Consumption in Adolescents

### 4.1. Beverage Choices and Water Intake Patterns

From the perspective of clinical dietetics and public health, one particularly concerning trend is the low intake of plain drinking water among adolescents, alongside the high consumption of SSBs, energy drinks, and carbonated soft drinks. International studies conducted as part of the Liq.In7 project have shown that in many countries, including those in Central Europe, water accounts for less than half of adolescents’ total fluid intake [24,51]. Research conducted among Chinese children and adolescents aged 6 to 17 years indicates that older children, boys, urban residents, and those from higher socioeconomic backgrounds were more likely to consume SSBs. Children who consumed SSBs 1 to <5 times per week (11.7%) or more than 5 times per week (12.9%) were more likely to be overweight or obese compared to those who consumed SSBs less than once per week [52]. In the United States, Leung [53] analyzed data from children aged 2–18 years collected as part of the National Health and Nutrition Examination Survey (NHANES) and found that children who consumed SSBs had poorer overall diet quality and higher total energy intake compared to those who did not consume SSBs. The authors suggest that interventions aimed at treating obesity and chronic diseases should focus on replacing SSBs with water, and on improving other aspects of diet quality that are correlated with SSB consumption. A study conducted by Kostecka [27] among Polish adolescents aged 11–13 years aimed to assess the hydration status of school-aged children and examine changes in the types and amounts of beverages consumed between 2018 and 2023. The findings indicate that, overall, fluid intake did not meet recommended dietary guidelines. The author notes that low fluid consumption may have a negative impact on hydration status and overall physiological functioning. In addition, consumer behavior analysis highlighted that taste preferences and advertising were strongly associated with higher intake of both carbonated and non-carbonated SSBs, as well as flavored milk drinks. The proportion of children purchasing drinks independently and having access to SSBs increased significantly over the study period. Another important issue raised by the author is the association between high sugar intake, particularly added sugars from beverages, and decreased attention and concentration in children, including those diagnosed with ADHD. Other Polish studies among adolescents aged 10–12 years have reported statistical analysis revealing that boys consume SSBs and energy drinks more frequently than girls [10]. Similar findings were reported by Błaszczyk-Bębenek [54], who found that energy drink (ED) consumption among children and adolescents in the Małopolska region was gender-dependent, with boys consuming significantly more EDs than girls and being more likely to purchase them in larger packaging formats. These findings are consistent with observations from other European countries. For example, in Germany and Spain, average daily water consumption among adolescents ranges from 1.1 to 1.3 L, which is below EFSA recommendations [22,55]. Of particular concern are the trends indicating earlier initiation of energy drink consumption. Studies from the United Kingdom and Scandinavian countries show that 15–20% of secondary school students consume energy drinks at least once per week, often in the morning, as a substitute for breakfast. Due to their caffeine and taurine content, such beverages have diuretic effects that may exacerbate dehydration and are also associated with irritability, sleep disturbances, and difficulty concentrating [56,57].

### 4.2. Availability and Access to Water in the School Environment

One of the key physiological changes during puberty is the increased secretion of sex hormones—estrogen and progesterone in girls, and testosterone in boys—which significantly influence mechanisms regulating fluid balance. Estrogen, which rises significantly in girls, has been associated with increased sodium and water retention, potentially leading to fluctuations in hydration status throughout the menstrual cycle [39,58]. A study conducted among a Chinese population of adolescent boys found that those who met recommended fluid intake levels had, on average, 1.3 kg more skeletal muscle mass (SMM), 0.9 kg higher intracellular water (ICW), and a 0.5% greater TBW/BW ratio compared to those who did not meet hydration recommendations (*p* < 0.05) [59].

Physiological differences between sexes become particularly pronounced during puberty and have significant implications for fluid and electrolyte balance. Girls tend to have a higher percentage of body fat, which corresponds to a relatively lower total body water content [60]. Moreover, cyclical hormonal fluctuations may influence fluid distribution and retention, as well as thirst perception during different phases of the menstrual cycle [36]. In contrast, boys generally exhibit a higher resting metabolic rate and greater levels of physical activity, which lead to increased heat production and sweat loss [60]. Studies suggest that boys are more likely to begin physical activity in a state of subclinical dehydration, which may impair performance and thermoregulation. Sex-based differences are also evident in beverage consumption behaviors; girls are more likely to choose flavored drinks such as coffee and sweetened tea, while boys more frequently consume energy and isotonic drinks [61]. A key factor influencing actual water consumption during the school day is the physical and social availability of drinking water. Although legislation in most European countries requires that students have access to drinking water, in practice, students often lack the opportunity or do not feel comfortable drinking water in the classroom. The *Water First* program study showed a reduction in water and SSBs intake between the 7th and 15th month of observation. Although students in the intervention group had access to water points throughout the study period, lessons and promotional activities ended after six months. The decline in intervention effects suggests the need for school leaders and consistent support personnel. The *Water First* program helped prevent an increase in the prevalence of overweight, though not obesity, among primary school students. Nonetheless, continued promotion of high-quality water sources in the school environment remains necessary. Studies conducted in secondary schools in the United States and Canada demonstrated that installing modern water dispensers and educating students about hydration led to an increase in water consumption by over 30% within a few months [62].

### 4.3. Peer Influence and Social Norms

Fluid consumption behaviors in adolescents are strongly influenced by peer dynamics. A study conducted by Dutch researchers analyzed the process of motivating the school community by selecting a subgroup of influential students and training them as “influence agents” to promote water consumption as an alternative to sugary soft drinks. The aim was to facilitate the diffusion of target behaviors among peers during the implementation of the intervention through social networks. The study outcomes highlight the significant role of peers and the school environment in supporting water consumption instead of SSBs [63]. Research suggests that adolescents often do not perceive drinking water as a biological need, but rather as a socially conditioned behavior. A qualitative study conducted by Wang [32] showed that the consumption of SSBs and sweet snacks is shaped not only by intrapersonal factors, but also by interpersonal, environmental, and social influences. Further consideration of environmental factors is supported by the observations of Kruitwagen van de Gaar [64], who studied children aged 6 to 13 years. Researchers examined the relationships between family and household factors and children’s consumption of SSBs. The findings indicate that the child’s age, parental attitudes, parents’ subjective norms, availability of SSBs at home and school, parenting practices, and parental modeling were all associated with children’s average daily intake of SSBs, measured in liters.

### 4.4. Family Environment and Parental Modeling

Significant positive correlations between parental practices/modeling and children’s SSB consumption highlight the important role parents play in shaping their children’s dietary habits. Parents serve as both role models and direct influencers of children’s diets. In a separate longitudinal study conducted in Poland, it was observed that consumer preferences remained the main criterion for beverage selection in both 2018 and 2023. Advertising was identified as a key determinant of beverage choice. The author reported that this determinant gained greater significance among consumers, rising from 52.1% in 2018 to 58.5% in 2023 (*p* < 0.05) [27].

### 4.5. Advertising, Media Exposure, and Digital Marketing

According to Remedios [65], SSBs are heavily marketed to children and adolescents, and the promotion of such products contributes to the consumption of unhealthy foods among youth aged 10–17 across six countries. The researchers employed an innovative method to assess beverage appeal using an emoji-based self-rating scale by asking the question: *“How much would you like to have this drink?”* The study found that more frequent exposure to marketing of specific beverage brands was associated with more positive attitudes toward those products, as well as greater brand recognition and acceptability. The promotion of food-related brands on social media has become an increasingly prominent marketing strategy targeting adolescents. A cross-sectional online study conducted among U.S. adolescents aged 13–17 examined their interactions with restaurant, food, and beverage brands they had ever liked, shared, or followed on social media. The results showed that 70% of teens reported interacting with at least one food or beverage brand on social media. Approximately half of the respondents reported engaging with fast food brands (54%), SSB brands (50%), candy brands (46%), and snack brands (45%), whereas only 7% reported interacting with brands in all other food and beverage categories [66].

## 5. Approaches to Improve Hydration

### 5.1. Increased Fluid Requirements During Physical Activity

The World Health Organization’s Global Action Plan on Physical Activity 2018–2030 was released in 2018. All 194 WHO Member States agreed to a new global target of a 15% relative reduction in physical inactivity by 2030 and called on the WHO to update the 2010 Global Recommendations on Physical Activity for Health [67]. Physical activity, both within and outside of the school setting, is one of the primary factors contributing to increased fluid loss. Individuals engaging in prolonged and intense exercise experience significant losses of body fluids due to thermoregulatory sweating. If these losses are not adequately replenished, endurance performance may deteriorate as a result of numerous physiological disruptions, including hyperthermia, hyperventilation, increased cardiovascular strain with reduced blood flow to the brain, skeletal muscles, and skin, greater reliance on muscle glycogen and cellular metabolism, alterations in neural activity, and in some cases, impaired muscle metabolism and aerobic capacity [68].

Sweating during physical exercise can lead to water loss ranging from 0.5 to as much as 2.5 L per hour, depending on exercise intensity and ambient temperature [69]. In adolescents, thermoregulatory capacity is not yet fully developed, resulting in less efficient cooling mechanisms and greater susceptibility to heat stress [3]. Studies conducted among high school students have shown that a significant proportion of adolescents begin physical education classes in a state of suboptimal hydration, as confirmed by urine osmolality and specific gravity measurements [70]. Chronic fluid deficits, when combined with repeated physical activity, can lead to reduced aerobic capacity, impaired motor coordination, and an increased risk of injury [34]. Through sweating, physically active individuals lose both water and electrolytes, particularly, sodium and chloride, as well as smaller amounts of potassium. During physical activity, electrolytes are essential because they serve various biological functions. In particular, sodium and potassium help regulate total body water. Sodium contributes to muscle excitability and cellular permeability, while potassium plays a role in the synthesis of proteins and carbohydrates. Chloride helps maintain osmotic pressure and acid–base balance and is a key component of gastric juice. In addition to water, which is a natural and universal hydration fluid, it is also worth considering the role of sports drinks. These beverages play an important role in rehydration, enhancing athletic performance, and supporting health in certain physiological conditions. Their formulations may be specifically designed to boost energy and improve focus. In the context of sports, the primary function of such beverages is to rehydrate and replenish electrolytes, carbohydrates (CHO), and other nutrients that may be depleted during exercise [71].

### 5.2. Hydration, Cognitive Function, and Academic Performance

Hydration is a fundamental determinant not only of physical health, but also of cognitive and psychological performance, particularly, during developmental stages. From the perspective of nervous system physiology, water is essential for maintaining proper osmotic pressure of cerebrospinal fluid, plasma volume, and cerebral blood flow [15,16,17]. Water acts as the medium for nerve impulse conduction, facilitates sodium–potassium ion exchange, and its deficiency leads to immediate disruptions in neuronal homeostasis. In children and adolescents, whose brains are still undergoing structural and functional development, the effects of inadequate hydration may be particularly significant and multidimensional [72].

This makes adolescents more vulnerable to fluid and electrolyte imbalances [28,40,41]. Even relatively mild dehydration can result in decreased blood pressure, reduced cerebral perfusion, and consequently, tissue hypoxia and limited glucose availability for neurons Functional MRI (fMRI) studies conducted in healthy adolescents have shown that dehydration leads to reduced activity in the prefrontal cortex—an area responsible for attention, planning, and decision-making—and increased activation in brain regions involved in processing negative emotions, such as the hippocampus and amygdala. These changes are correlated with symptoms such as irritability, difficulty concentrating, and declines in mood and well-being [73,74,75]. Intervention studies involving children and adolescents suggest that consuming even small amounts of water (200–500 mL) can result in significant improvements in neurocognitive test performance—including short-term memory, sustained attention, reaction time, and logical reasoning [16,46]. In a randomized controlled trial conducted by Fadda [45], children aged 9–12 years who consumed water prior to cognitive testing achieved higher scores in attention and spatial memory tasks compared to the control group.

**Figure 2:**
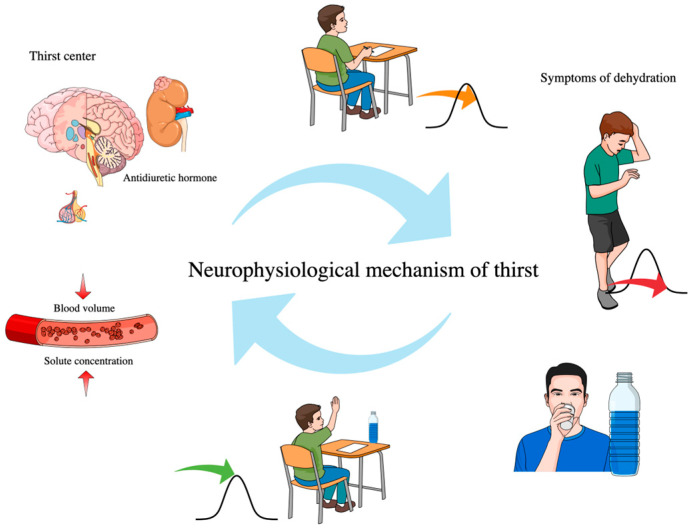
Neurophysiological mechanism of thirst, its relation to dehydration, and impact on cognitive functioning in adolescents. The diagram illustrates the physiological pathway from dehydration to reduced cognitive performance: a decrease in plasma volume and increase in electrolyte concentration activate the thirst center and stimulate antidiuretic hormone release, ultimately triggering the sensation of thirst and reactive fluid intake. Among adolescents, this response is often delayed; they tend to drink fluids only after early symptoms of dehydration appear. This delay may impair cognitive functions such as concentration, mood, and decision-making.

In the educational context, hydration plays a critical role in the learning process and knowledge acquisition. Adequate fluid intake supports perception, comprehension, analysis, and memory formation. A dehydrated brain has a reduced capacity to process stimuli, which directly affects learning outcomes and academic performance (Figure 2) [72]. In classroom settings where students are required to maintain attention for several hours, often with limited physical movement and restricted access to fluids, even mild dehydration can have cumulative effects [43,45]. From a dietetic and public health perspective, implementing systemic solutions in schools is essential. These include providing free access to drinking water in hallways and classrooms, allowing students to drink water during lessons, incorporating education on the role of water in daily life and cognitive processes, and monitoring fluid intake using validated tools such as the BEVQ-15 questionnaire, which enables assessment of both the quantity and quality of beverage consumption. Such initiatives not only promote student health but may also contribute to improved academic performance and learning outcomes [76,77,78]. To effectively address suboptimal fluid intake among adolescents, comprehensive intervention strategies are required-encompassing environmental, educational, and social factors. Based on a review of the literature, this section outlines the most commonly implemented approaches, whose effectiveness has been supported by studies conducted across different countries and school populations. The table (Table 3) below summarizes these key strategies, including a brief description, observed outcomes, and relevant references. It is intended as a practical tool to guide the development of future school- and community-based interventions aimed at improving hydration status in adolescents.

### 5.3. Critical Analysis of Recommendations and Implementation Barriers

It is important to note that there are significant methodological differences between existing guidelines. The EFSA bases its recommendations primarily on water balance studies and intake analysis across EU countries, while the IOM determines requirements based on energy expenditure (which does not account for variations in sweating, climate, or dietary water content). Additionally, different institutions use varying definitions of “total fluid intake”; some include water from food, while others count only beverages. Moreover, there is a lack of effective population-based tools for monitoring the quantity and quality of beverage intake. Most studies rely on general dietary questionnaires that do not accurately reflect the types of fluids consumed. In this context, the BEVQ-15 questionnaire may offer a promising solution, as it enables both volumetric and qualitative assessment of fluid intake in population studies [72].

The effectiveness of hydration recommendations largely depends on their implementation in practice. Patel [62] demonstrated that students in schools where educational programs on hydration were introduced showed improved hydration levels. Current guidelines are necessary but insufficient; they require updating to incorporate environmental, behavioral, and sex-specific factors. It appears essential to develop hydration standards tailored specifically to school-aged adolescents, taking into account their actual physiological and environmental conditions. Moreover, tools for monitoring hydration status, including validated methods and mobile applications, should be developed and standardized. Equally important is the integration of hydration guidelines into school practice through education, ensuring water availability, and eliminating social barriers [76,77].

Ensuring adequate hydration among adolescents in the school environment is not merely a matter of access to water; it is a complex structural, social, and educational issue that requires a systemic approach.

Although numerous public health institutions emphasize the importance of hydration for student development and cognitive functioning [67], many barriers still limit water consumption in practice. These have been identified at infrastructural, institutional, environmental, and individual levels [10,47,78]. Despite the availability of scientific knowledge regarding the effects of hydration on attention and academic performance, education in this area remains insufficient. Biology and physical education curricula, as well as preventive health campaigns, rarely address water as a critical dietary component [79,80]. Qualitative studies reveal that many students are unable to correctly indicate the recommended daily water intake or recognize symptoms of dehydration [81]. Similarly, parents—who serve as role models for dietary behavior—are often unaware of the consequences of insufficient water consumption in adolescents, including both physical health effects (constipation, headaches, fatigue) and mental functioning (irritability, decreased attention, anxiety) [82,83].

### 5.4. Research Gaps and Future Research Needs

Although the role of water in maintaining health and cognitive functioning in adolescents is well established theoretically, there remain several areas where current knowledge is limited, inconsistent, or requires verification within specific cultural and environmental contexts. Determining actual fluid needs, assessing real behaviors of adolescents in school environments, and understanding behavioral and structural barriers all call for further targeted research. One of the main research gaps is the lack of current, representative population-level data on hydration status in adolescents, especially within the European and Central European contexts. Much of the available evidence comes from the United States or Asia and is often based on reviews or secondary data analyses. Another key issue is the lack of standardization in tools for assessing fluid intake. Questionnaires used in studies frequently fail to distinguish between water, functional beverages, juices, and energy drinks. As a result, comparing findings across countries or age groups becomes difficult. Moreover, regarding tools tailored to school-aged youth, there is a shortage of validated tools that are both sensitive and practical for use in school settings [78,84,85,86,87,88].

Most of the available research on adolescent dehydration focuses on short-term effects such as its impact on concentration, fatigue, physical performance, or mood. However, little is still known about the long-term consequences of chronic, mild dehydration during adolescence. Potential outcomes such as impaired glucose metabolism, increased risk of overweight, insulin resistance, and reduced cognitive function in adulthood require further cohort and prospective studies. Research on nervous system functioning suggests that chronic dehydration may exacerbate depressive symptoms, anxiety, and even impair brain neuroplasticity during the developmental stage, although evidence in this area remains limited [43].

## 6. Challenges and Future Directions

### 6.1. Critical Review of Current Recommendations

Despite a growing number of institutional guidelines for fluid intake in adolescents (EFSA, IOM, AAP, NIH), practical implementation remains inconsistent. These recommendations differ in methodology; some are based on water balance studies, while others rely on energy expenditure calculations. However, they often overlook crucial contextual variables such as climate, physical activity, menstrual cycle-related hormonal fluctuations, or caffeine consumption. Moreover, there is a lack of consistency in the definition of total water intake; some guidelines include fluids from food, while others consider only beverages. This lack of standardization hinders both implementation and comparative research.

### 6.2. Barriers to Implementation

Effective hydration in adolescents is not merely a physiological concern—it is a structural and behavioral issue. Schools are key environments in this regard, yet many face infrastructural and regulatory limitations. Water fountains, dispensers, or even bottled water access are often lacking or restricted. Short class breaks and informal norms discourage water consumption during lessons. Furthermore, students are heavily influenced by peer behaviors and aggressive marketing of SSB, which often undermine health-oriented choices. Even when students are aware of hydration recommendations, environmental and social barriers may prevent them from acting accordingly. Educational policies also fall short. Hydration is rarely integrated into biology or physical education curricula, and national health promotion campaigns often ignore water as a key dietary element. Studies reveal that many students cannot identify recommended water intake levels or recognize early symptoms of dehydration. Parents, as primary role models for dietary behaviors, are often unaware of the cognitive and physical consequences of inadequate hydration, ranging from constipation and fatigue to anxiety and decreased academic performance.

### 6.3. Research Gaps and Future Needs

Several areas of adolescent hydration research remain underexplored. The majority of available data originate from the USA and Asia, with limited evidence from Central and Eastern Europe, including Poland. This geographical imbalance hinders the formulation of culturally and environmentally relevant public health strategies. There is also a notable lack of validated, population-appropriate tools to monitor fluid intake and hydration status among school-aged youth. Many dietary questionnaires do not differentiate between plain water, functional beverages, juices, and energy drinks. As a result, cross-national comparisons and policy assessments remain problematic. Tools like the BEVQ-15 show promise but require further validation across different age groups and cultural settings. Moreover, existing research predominantly focuses on the short-term consequences of dehydration such as decreased attention, fatigue, or impaired physical performance. Much less is known about the long-term effects of chronic, mild dehydration during adolescence. Emerging evidence suggests potential links to impaired glucose metabolism, insulin resistance, increased risk of obesity, and diminished neuroplasticity, but longitudinal studies are lacking.

## 7. Conclusions and Recommendations

Although research clearly confirms that even mild dehydration affects cognitive function in adolescents, this issue remains marginalized in public health policies and educational systems. A consistent pattern emerges across the analyzed publications: students regularly fail to meet recommended fluid intake levels. Importantly, the problem lies not only in the quantity of fluids consumed, but also in the environmental and psychosocial context in which these choices are made.

Educational messages promoting water as the healthiest beverage cannot be effective when schools do not provide functional water sources, breaks are too short, and drinking during lessons is formally prohibited.

At the same time, existing institutional guidelines (EFSA, AAP, IOM, NIH) offer precise reference values for fluid intake yet differ in their consideration of contextual variables such as physical activity intensity, caffeine intake, or hormonal fluctuations during the menstrual cycle. Moreover, most policy documents lack reference to implementation barriers within school settings, such as infrastructure, internal regulations, peer pressure, or habits shaped by the marketing and social media presence of SSBs.

The reviewed literature highlights the need to approach adolescent hydration not solely as a physiological issue, but rather as a complex biopsychosocial phenomenon, influenced by structural, behavioral, and cultural factors.

Quantitative studies point to fluid deficits and their cognitive consequences, while qualitative studies, although much less common, indicate perceptual barriers. Importantly, the majority of available data come from Anglo-Saxon and Asian countries, while context-specific research grounded in Central and Eastern European settings, including Poland, is scarce and fragmented. This hinders the development of locally relevant recommendations and the implementation of political action.

Importantly, existing intervention models combining improved access to drinking water and educational activities have demonstrated measurable effects on students’ hydration levels and cognitive performance, suggesting a scalable pathway for public health practice.

This integrative review juxtaposing physiological data with environmental and psychosocial analyses suggests that only an interdisciplinary approach can truly reflect the complexity of the hydration problem among school-aged youth. It thus offers a proposal for a theoretical framework for analysis and intervention that addresses not only the question of how much adolescents drink, but more importantly, why they don’t and how this can be improved. The key messages of the review are summarized in Table 4, which outlines the most important conclusions regarding hydration compliance, the consequences of underhydration, behavioral determinants, and effective intervention strategies.

## Figures and Tables

**Table 1:** Comparison of recommended total water intake (TWI) values published by European and American health authorities.

		EFSA (2010) [18]	IOM (2004) [19]
Sex	Age	mL/Day *	mL/Day *
Girls Boys	9–13	1900	2100
	9–13	2100	2400
Female adolescents Male adolescents	14–18	2000	2300
	14–18	2500	3300

* Values refer to total water intake (TWI) = plain water + beverages + water from food.

**Table 2:** Adequate Intake (AI) recommendations for total water intake by the National Institute of Public Health—NIH (formerly the Institute of Food and Nutrition, Warsaw).

Sex	Age (Years)	AI (mL/Day) *
Girls Boys	10–12 10–12	1900 2100
Female adolescents Male adolescents	13–15 13–15	1950 2350
Females Males	16–18 16–18	2000 2500

* Values refer to total water intake (TWI) = plain water + beverages + water from food.

**Table 3:** Summary of Interventions Aimed at Improving Hydration in Adolescents.

Type of Intervention	Description	Observed Outcome	Source
School infrastructure	Installation of water dispensers, access to water during classes	↑ Water consumption, ↓ SSBs intake, improved hydration markers	[62]
Educational programs	Lessons about hydration, posters, hydration diaries	↑ Knowledge, ↑ self-reported fluid intake	[45]
Peer-led interventions	“Influence agents” promoting water instead of sugary drinks	↑ Peer support, ↑ social acceptance of water consumption	[63]
Media and social marketing	Emoji-based appeal tests, exposure to ads, social media campaigns	↑ SSB preference, ↑ brand recognition, ↓ water preference	[65]
Monitoring tools	Use of BEVQ-15 questionnaire in school programs	Better tracking of beverage choices, awareness of intake patterns	[76]

**Table 4:** Key Messages: Adolescent Hydration.

Area	Key Insight
1. Hydration Compliance	Up to 75–85% of adolescents fail to meet daily water intake recommendations across different countries (e.g., Poland, Germany, UK, China).
2. Main Consequences	Even mild dehydration is associated with reduced attention, impaired memory, irritability, fatigue, and lower physical performance.
3. Determinants of Behavior	Key factors include school water access, peer influence, parental modeling, and aggressive marketing of sugary drinks.
4. Effective Strategies	Interventions combining water infrastructure improvements and educational activities show measurable effects on hydration and cognition.

## Data Availability

The original data presented in the study are openly available in PubMed.

## Abbreviations

The following abbreviations are used in this manuscript:

EFSA	European Food Safety Authority
IOM	Institute of Medicine
SSB(s)	Sugar-sweetened beverage(s)
SMM	Skeletal muscle mass
ICW	Intracellular water
TBW/BW	Total body water/body weight
RAA	Renin–angiotensin–aldosterone system
AVP	Arginine vasopressin
V2	Vasopressin type 2
HPA	Hypothalamic–pituitary–adrenal axis
fMRI	Functional magnetic resonance imaging
BEVQ-15	Beverage Intake Questionnaire (15-item)
AI	Adequate intake
TWI	Total water intake
WHO	World Health Organization
AAP	American Academy of Pediatrics
NHANES	National Health and Nutrition Examination Survey
NIH	National Institute of Public Health
ADHD	Attention deficit hyperactivity disorder
EU	European Union
ED	Energy drink
CHO	Carbohydrates
Liq.In7	Liquid Intake 7-day record project
mOsm/kg	milliosmoles per kilogram
